# Stoma reversal after emergency stoma formation—the importance of timing: a multi-centre retrospective cohort study

**DOI:** 10.1186/s13017-025-00598-3

**Published:** 2025-03-29

**Authors:** Scott MacDonald, Anna Gallagher, Lauren McNicholl, Luke McElroy, Rebecca Hughes, Tara Quasim, Susan Moug

**Affiliations:** 1 Department of Surgery, Royal Alexandra Hospital, Paisley, Scotland; 2 Department of Surgery, Golden Jubilee University National Hospital, Clydebank, Scotland; 3 Department of Anaesthesia, Pain and Critical Care, University of Glasgow, Glasgow Royal Infirmary, Glasgow, Scotland

**Keywords:** Emergency, Stoma, Reversal, Complications, Ileostomy, Colostomy, Jejunostomy, Hartmann’s

## Abstract

**Background:**

Restoration of intestinal continuity is a key consideration for patients having a stoma created under emergency conditions. There is contrasting evidence about the outcomes of stoma reversal for these patients. This research aims to describe the post-operative outcomes of stoma reversal after emergency formation, and whether these are affected by the timing of reversal.

**Methods:**

A retrospective review of a prospectively maintained emergency laparotomy (EmLap) database for 4 hospitals was performed between 2018 and 2021. Adult patients undergoing emergency stoma formation were identified and followed up until 2024. Those undergoing stoma reversal surgery were included in the final analysis. A Cox proportional-hazards model was created to identify factors associated with increased time to reversal.

**Results:**

1775 patients had an EmLap, with 505 (28.5%) having a stoma created. Of those patients with a stoma, 97 patients (19.2%) died within one year post-operatively. 146 (28.9%) of the emergency stoma patients underwent stoma reversal, with median time to reversal of 16.9 months. Median post-operative length of stay was 7 days, and 52.1% of patients sustained complications within 30 days post-operatively. Patients reversed within 18 months of stoma formation had fewer significant complications (7.9% v 35.1%, p < 0.001), a shorter length of stay (6 days v 7 days, p < 0.001), and reduced post-operative ileus rates (21.3% v 64.9%, p < 0.001) than those reversed after this period. Receiving adjuvant therapy for malignancy (adjusted Hazard ratio 0.36, 0.17–0.78, p = 0.001) and being male (adjusted Hazard ratio 0.69, 0.49–0.97, p = 0.032) were significantly associated with increased time to reversal.

**Conclusion:**

Emergency stoma formation is commonly performed during EmLap, but the majority of emergency stomas are never reversed. The complication profile for reversing these stomas is significant, but early reversal is associated with better post-operative outcomes. Standards of care for emergency stoma patients would be welcome in order to improve outcomes for this cohort.

## Background

There are approximately 20,000 new intestinal stomas formed annually in the UK, with 1 in 5 of these being formed in the emergency setting [1]. These emergency stoma patients have high rates of stoma-related complications (SRCs), such as skin excoriation, para-stomal herniae or stoma retraction, with recent work reporting that over 75% will develop SRCs [2]. With reduced quality of life in comparison to patients with a stoma formed in the elective setting [3], restoration of intestinal continuity is often a key consideration for these patients [4].

Current stoma reversal rates after emergency formation are unclear, with variation in the surgical literature explained by factors such as stoma type and geographical location of the healthcare provider. For example, permanent stoma rates after an emergency sigmoid colectomy and end colostomy (Hartmann’s procedure) have a reported variation of 20% to 70% [5–8], predominately derived from data from the United States, the Netherlands or Scandinavia. In contrast, there is limited data on reversal of an emergency stoma from within the National Health Service (NHS) in the United Kingdom (UK). The structure of the nationally funded health-care system within the UK may result in differences in provision of elective surgery, as well as baseline differences in patient profile, compared to other health-care systems.

Research regarding post-operative outcomes for emergency stoma patients under-going restoration of intestinal continuity is sparse, but the available data suggests significant complication rates of between 3.7 and 20.5% [9–11]. These studies are limited in that they frequently fail to differentiate emergency stoma patients from those who have had their stoma created under elective conditions. Additionally, the vast majority of data focusses on stoma reversal after an emergency sigmoid colectomy and end colostomy, meaning there is a substantial gap in knowledge for patients with other emergency stoma types, such as end ileostomies.

With regards to timing of reversal, it has been shown that early reversal of an elective ileostomy appears to be safe, feasible, and may reduce the incidence of SRCs for patients [11–13]. However, there is a lot of variation in implementing this into clinical practice for elective stoma patients [14]. The only guidelines available within the UK regarding timing of stoma reversal are produced by the National Bowel Cancer Audit (NBOCA) who outline an 18-month cut-off as a Key Performance Indicator target for stoma reversal for elective colorectal cancer patients included in their database [15]. No specific guidance is available for the emergency stoma cohort at present, although it has been shown that the time to reversal for emergency stoma patients is often longer than their elective counterparts [10, 16]. Additionally, there is much scarcer evidence comparing the difference in post-operative outcomes in relation to timing of reversal for emergency stoma patients. Therefore, the guidance for patients and surgeons regarding optimal timing of reversal is currently lacking.

The primary aims of this research are to identify the incidence of stoma formation during EmLap and to document those undergoing reversal. The secondary aims are to describe the post-operative outcomes of stoma reversal, whether these are affected by the timing reversal and to identify any risk factors for delayed reversal.

## Methods

Consecutive patients undergoing formation of an intestinal stoma under emergency conditions (as per National Emergency Laparotomy Audit (NELA) criteria [17]) across four acute NHS hospital sites were included. Patients were identified from a retrospective review of a prospectively maintained EmLap database (Emergency Laparotomy and Laparoscopic Scottish Audit–ELLSA) for the four sites from 1st January 2018 until 31st December 2021 (48 months). The retrospective review was performed in June 2024. ELLSA is a Scottish Government initiative supported by the Modernising Patient Pathways Programme [18].

### Ethical approval

Data collection for ELLSA is covered by pre-existing Caldicott Guardian approval, therefore formal ethical approval for this project was not required as it involved retrospective analysis of pre-collected data for ELLSA.

This manuscript has been reported in keeping with the STROBE guidelines for observational studies [19].

### Inclusion/exclusion criteria

Patients were included in the ELLSA database if they had an EmLap meeting NELA criteria [17]. Patients identified from this database who underwent the creation of a new intestinal stoma (i.e. an ileostomy, colostomy or jejunostomy) were included in this study.

As per NELA exclusion criteria, those patients who had a stoma created purely for the purposes of feeding (e.g. a gastrostomy or feeding jejunostomy), the creation of a urinary stoma (urostomy or nephrostomy) or underwent laparoscopic formation of a defunctioning stoma with no further procedure performed were excluded.

### Data collection

Demographic data collected included: patient age; sex; socio-economic deprivation status (as defined by the Scottish Index of Multiple Deprivation (SIMD) [20]); and pre-operative frailty status (Rockwood Clinical Frailty scale [21], with a score of 5 or greater indicating those living with frailty).

Clinical data were collected from the patients' index emergency stoma formation and included: smoking status; presence of cardiovascular disease, respiratory disease, diabetes, or immunosuppression; Body Mass Index (BMI); American Society of Anaesthesiologists score (ASA); indication for emergency stoma formation; procedure performed; type of stoma created; length of hospital stay; requirement for intensive care admission; 30-day complication rates; rate of major complications (i.e. Clavien-Dindo score ≥ 3 [22]); 30-day and overall mortality rates. Multi-morbidity was defined as the presence of more than one of: cardiovascular disease; respiratory disease; diabetes; or immunosuppression.

Clinical data collected after stoma reversal included: timing of reversal in comparison to index stoma formation; length of hospital stay; 30-day complication rates; 30-day mortality rate; and days taken until the GI-2 composite outcome was achieved.

The validated GI-2 composite outcome has previously been used as a post-operative outcome measure for patients undergoing gastrointestinal surgery, and is defined as the time taken in days to pass first stool and tolerate oral intake [23, 24].

### Follow-up

Patients were followed up until June 2024, ensuring that each patient had a minimum of 30 months follow-up after their index emergency stoma formation. The decision of whether to pursue stoma reversal was determined on a case-to-case basis between the individual surgeon and patient, as there are currently no clinical guidelines available to standardise this decision.

Patients who were lost to follow-up within one calendar year post-operatively were excluded from analysis. Those who died within one calendar year post-operatively were not included in any analysis regarding factors associated with delayed time to reversal or stoma non-reversal.

### ‘Standard’ and ‘delayed’ reversal

An interval of less than 18 months after index stoma formation was defined as representing a ‘standard’ reversal period for emergency stoma patients by this group of authors, in keeping with the NBOCA guidelines [15].

### Missing data

Any data missing from the ELLSA database was input manually by the lead researcher by retrospective analysis of the patient’s electronic clinical records.

Frailty score was only routinely documented for patients who were 65 years old or older as per current ELLSA guidelines [18].

### Statistical analysis

Normally distributed continuous data were expressed as mean and standard deviation (SD). Non-parametric continuous data were expressed as median and inter-quartile range (IQR). Means were compared using the students t-test and medians were compared using the Mann–Whitney U test. Categorical data were compared using the Chi-square test. Survival data were compared graphically using a Kaplan–Meier chart and statistically using the log-rank test. The specific test used for comparison has been specified on each occasion. A p-value of < 0.05 was considered statistically significant. 95% Confidence Intervals are reported when calculated. Statistical analysis was performed on SPSS (Statistical Package for Social Sciences, version 29, IBM).

For factors associated with an increased time period to stoma reversal, a Cox proportional-hazards model was created, utilising the following patient and clinical variables in a univariate format initially: age, sex, deprivation, frailty status, immunosuppression, cardiovascular disease, diabetes, respiratory disease, smoking status, ASA ≥ 3, BMI, presence of malignancy, requirement for adjuvant therapy, and stoma type. These factors were chosen for the model based on potentially significant relationships previously identified within the surgical literature [10, 25–27], and were agreed on by consensus of the authors a priori. Any factors identified as statistically significant from univariate analysis (i.e. p < 0.05), were taken forward to the final multi-variate Cox proportional-hazards model, to produce adjusted hazard ratios for increased time to stoma reversal.

A similar process was undertaken with the same pre-defined variables for factors associated with stoma non-reversal. These were utilised to create univariate logistic regression models initially, from which any factors that were statistically significant were taken forward to the final multi-variate logistic regression model, to produce adjusted odds ratios for stoma non-reversal in this cohort.

## Results

**Fig. 1:**
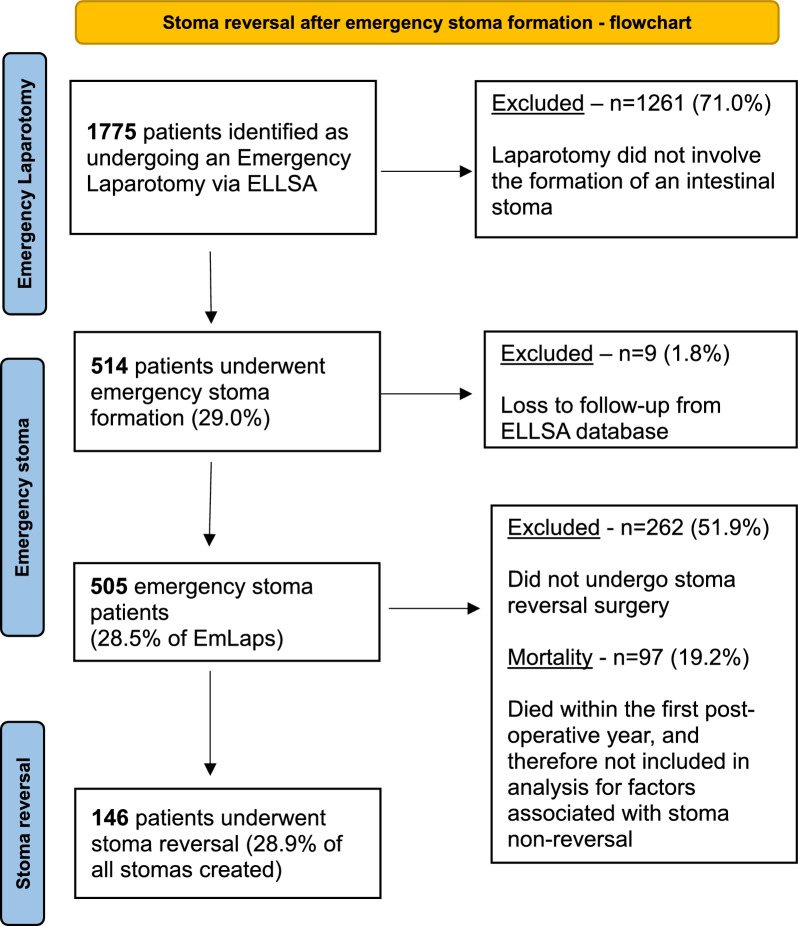
Flow chart of patient identification

A total of 1775 EmLap patients were identified during the 4-year study period. 9 patients (1.8%) were lost to follow-up from the ELLSA database and were excluded from analysis, leaving 505 emergency stoma patients for inclusion (28.5% of all EmLaps). 97 patients died within the first post-operative year (19.2%) (Fig. 1).

These patients were followed up for a median period of 35 months (IQR 19, minimum 30 months, maximum 78 months). 146 patients underwent reversal of their stoma during this follow-up period (28.9% of all emergency stomas created).

### Patient demographics: index emergency stoma formation

At index emergency stoma formation, patients had a median age of 63 years old (Range 18 -92 years, IQR 20 years) and 52.1% were male (Table 1). 34.5% of patients were living in the most deprived SIMD quintile. 220 patients (43.6%) in this cohort had underlying cardiovascular disease, 140 (27.7%) had respiratory disease, 122 (24.2%) were immunosuppressed and 75 (14.9%) had diabetes. 192 (38.0%) patients were living with multi-morbidity. The majority of this cohort had an ASA of 3 or greater (319 patients, 63.2%) and 70 patients of those with a frailty score documented (28.2%) were classified as living with frailty.

**Table 1:** Baseline patient demographics

	Total	Reversed		Not reversed*		p-value
		(n)	(%)	(n)	(%)	
	505	146	28.9	262	51.8	
Median follow up (Months, IQR)	35 (19)	36	(20)	34	(17)	0.826
Age (Years, IQR)	63 (20)	57.5	(19)	62.8	(19)	< **0.001**
Males	263	80	54.8	129	49.2	0.316
Most deprived SIMD quintile**	174	38	26.0	95	36.3	**0.030**
Immunosuppressed	122	27	18.4	73	27.9	**0.031**
Diabetic	75	17	11.6	41	15.6	0.254
CVD	220	48	32.9	101	38.5	0.231
Respiratory disease	140	39	26.7	67	25.6	0.835
Smoker	171	43	29.5	85	32.4	0.500
ASA ≥ 3	319	80	54.8	189	72.1	** < 0.001**
Obese (BMI ≥ 30)	120	40	27.4	61	23.3	0.378
Underweight (BMI ≤ 18.5)	46	10	6.8	28	10.7	0.191
Living with frailty (Rockwood Frailty Score > 4)***	70	19	15.3	37	29.8	** < 0.001**
Stoma type—Colostomy	227	68	46.6	118	45.0	0.923
Stoma type—Ileostomy	269	73	50	141	53.8	0.923
Stoma type—Jejunostomy	9	5	3.4	3	1.1	0.123

IQR—Interquartile range; SIMD—Scottish Index of Multiple Deprivation; CVD—Cardiovascular Disease; ASA—American Society of Anaesthesiologists score; BMI—Body Mass Index

Bold indicates p < 0.05

*Patients who died within one calendar year (n = 97) were not included in analysis for factors associated with stoma non-reversal

**The most deprived SIMD quintile is SIMD 1, which is the most deprived 20% of the Scottish population based on address and postcode

***Calculated total from the 248 older adult patients (48.2%) who had a formal frailty score documented

Of the stomas created, 269 were ileostomies (53.2%), 227 were colostomies (45.0%) and 9 were jejunostomies (1.8%). 447 end stomas (88.5%) and 58 loop stomas (11.5%) were formed.

**Fig. 2:**
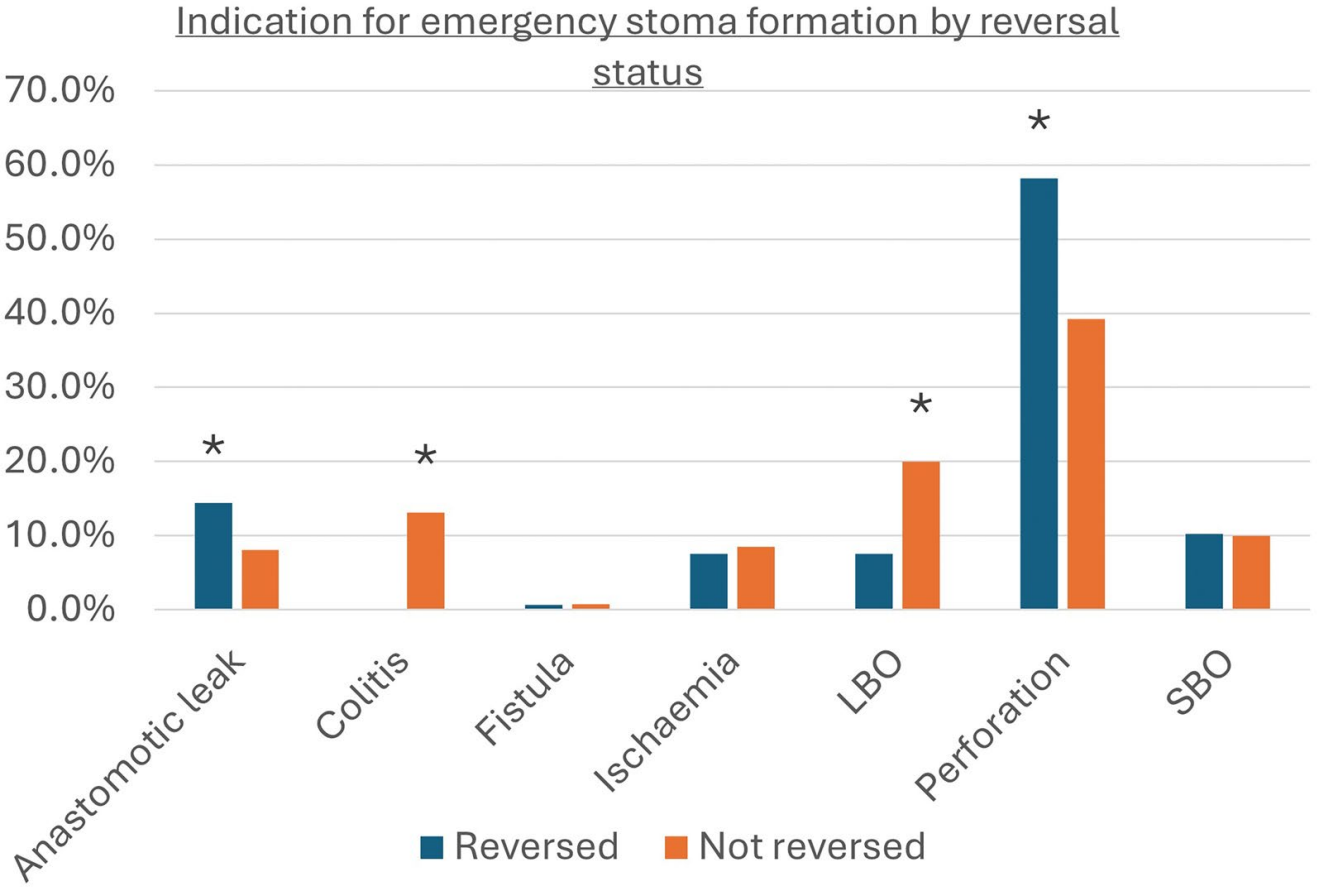
Aetiology for index emergency stoma formation. Asterisk (*) indicates statistically significant difference between reversed and non-reversed cohorts (p < 0.05)

The most prevalent indications for emergency stoma formation were gastrointestinal perforation (46.3%) and large bowel obstruction (15.9%) respectively (Fig. 2).

### Patient demographics: stoma reversal

The 146 patients (28.9%) who underwent stoma reversal had a median age of 57.5 years old (Range 23–92 years, IQR 15) and 55% were male. 50% of the stomas that were reversed were ileostomies. 129 end stomas (28.8% of total end stomas created) and 17 loop stomas (29.3%) were reversed.

The most common indications for index stoma formation in the stoma reversal group were gastro-intestinal perforation (58%), anastomotic leak (14%) and small bowel obstruction (10%) respectively (Fig. 2).

Those who underwent reversal were: younger (57.5 years v 62.8 years, p < 0.001); less likely to be living with frailty (15.3% v 29.8%, p < 0.001); less likely to be living in socio-economic deprivation (26.0% v 36.3%, p = 0.030); less co-morbid (54.8% ASA ≥ 3 v 72.1%, p < 0.001); and less likely to be immunosuppressed (18.4% v 27.9%, p = 0.031) than those who survived but did not undergo stoma reversal (Table 1).

Patients who underwent reversal were more likely to have had a shorter length of stay for their index emergency stoma formation (15 days v 19 days, p = 0.023), less likely to have required an intensive care unit admission (10.6% v 20.7%, p = 0.038) and less likely to have sustained significant complications at index procedure (i.e. Clavien Dindo grade III or IV—22% v 32%, p = 0.032).

### Outcomes from stoma reversal surgery

The median time to stoma reversal for the 146 patients was 16.9 months, with wide variation identified (range 1.6–52.7 months, IQR 16.2 months).

The median post-operative length of stay for these patients was 7 days (range 1–90 days, IQR 6 days). 76 patients (52.1%) sustained complications within 30 days post-operatively, with 38.4% of patients developing an ileus requiring the insertion of a naso-gastric tube. The stoma reversal cohort took a median of 3 days (range 1–10 days, IQR 3 days) to meet the composite GI-2 outcome (i.e. pass stool and tolerate oral intake).

In total, 18.5% of patients developed significant post-operative complications after their stoma reversal (i.e. Clavien-Dindo grade III or IV, and therefore requiring endoscopic, radiological or surgical re-intervention, or resulting in critical care admission). Additionally, 2 patients died within 30 days of their reversal procedure, giving a 30-day mortality rate of 1.4% for this elective procedure. There was no statistically significant difference in 30-day complications based on the primary operating surgeon grade (Trainee 58% v Consultant 43%, p = 0.079).

**Fig. 3:**
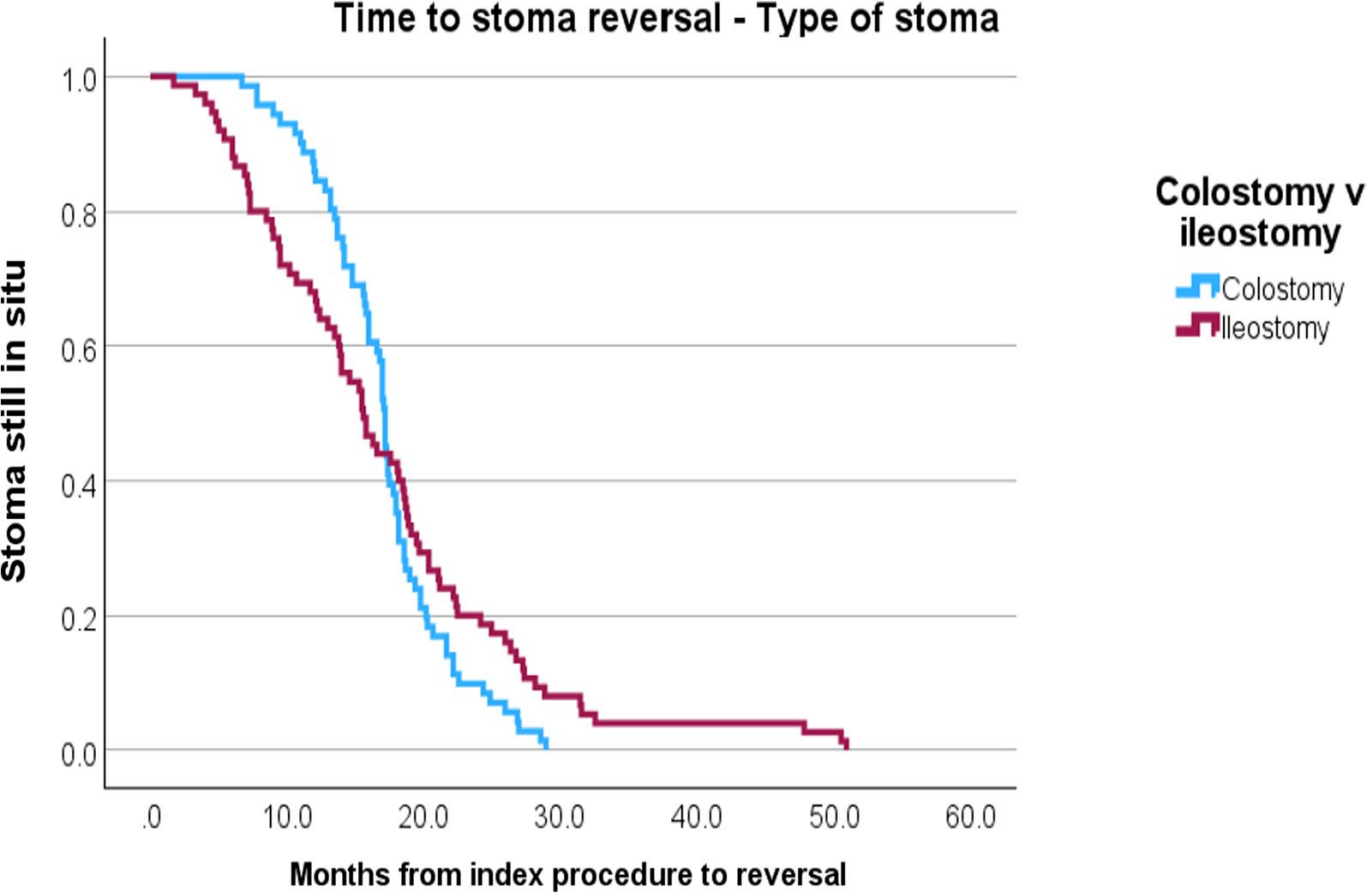
Kaplan–Meier survival curve for time to stoma reversal by stoma type

Those undergoing reversal of a colostomy were more likely to sustain 30-day complications compared to the ileostomy patients (60.3% v 43.8%, p = 0.040), but there was no statistical difference in significant complications (23.5% v 12.3%, p = 0.114). Length of hospital stay was similar between ileostomy and colostomy reversal groups (Median 6 days v 7 days, p = 0.285) (Table 2). There was no statistically significant difference between the stoma types in terms of timing of reversal (Median time to reversal for ileostomy 15.1 months v 16.7 months for colostomies, p = 0.351, log rank test) (Fig. 3).

**Table 2:** Ileostomy versus colostomy stoma reversal*—*post-operative outcomes

	Ileostomy		Colostomy		p-value
	(n)	(%)	(n)	(%)	
	73	50%	68	46.6%	
Time from index formation to reversal (Months, IQR)	15.1	11.8	16.7	5.2	0.351^*^
‘Delayed’	27	37.5%	30	44.1%	0.185
Length of stay (Days, IQR)	6	7	7	6	0.285
30-day complications	32	43.8%	41	60.3%	**0.040**
Clavien Dindo complications grade ≥ 3	9	12.3%	16	23.5%	0.114
Time until GI-2 achieved (Days, IQR)	3	2	3.5	2.5	0.384

Bold indicates statistically significant difference (p < 0.05); * = log-rank test for survival

### Standard versus delayed reversal

89 patients (61%) had their stoma reversed within 18 months of their index emergency stoma formation (i.e. a ‘standard’ reversal). These patients had a shorter post-operative length of stay (6 days v 7 days, p < 0.001), a reduced need for nasogastric tube insertion for post-operative ileus (21.3% v 64.9%, p < 0.001), and also a reduced time to reach the composite GI-2 outcome (3 days v 4 days, p < 0.001) than those reversed after this time period (Table 3). The ‘standard’ reversal group had reduced rates of 30-day complications, and less than a quarter of the significant complications that were seen in the ‘delayed’ reversal group (7.9% Clavien-Dindo ≥ 3 complications v 35.1%, p < 0.001).

**Table 3:** Standard v delayed stoma reversal—post-operative outcomes

	Standard reversal		Delayed reversal		p-value
	(n)		(n)		
	89	61.0%	57	39.0%	
Time from index to reversal (Months, IQR)	9.2	7.1	26	11.6	** < 0.001**
LOS (Days, IQR)	6	5.5	7	7	**0.018**
30-day morbidity	37	41.6%	39	68.4%	**0.009**
Clavien Dindo complications grade ≥ 3	7	7.9%	20	35.1%	** < 0.001**
NG required for post-operative ileus	19	21.3%	37	64.9%	** < 0.001**
Time until GI-2 achieved (Days, IQR)	3	1	4	1	** < 0.001**

Bold indicates statistically significant difference (p < 0.05)

### Factors associated with increased time to reversal

The results of the Cox proportional-hazards model for factors associated with increased time to stoma reversal can be seen in Table 4.

**Table 4:** Cox proportional hazards regression analysis for factors associated with delayed time to stoma reversal

Univariate analysis				Multi-variate analysis		
Factor	p-value	HR	95% CI	p-value	Adjusted HR	95% CI
Age	0.702	0.99	(0.98–1.02)			
Sex (Male v female)	**0.028**	0.83	(0.70–0.98)	**0.032**	0.69	(0.49–0.97)
Deprivation^1^	0.477	0.84	(0.53–1.35)			
Immuno-suppressed	0.095	0.70	(0.46–1.06)			
CVD	0.307	0.91	(0.77–1.09)			
Diabetes	0.617	0.94	(0.73–1.21)			
Respiratory disease	0.784	1.03	(0.85–1.23)			
Smoker	0.683	1.08	(0.75–1.57)			
ASA ≥ 3	0.484	0.91	(0.70–1.18)			
BMI	**0.043**	1.03	(1.00–1.07)	0.076	1.03	(0.99–1.06)
Malignancy	0.330	1.11	(0.90–1.36)			
Adjuvant treatment	**0.003**	0.31	(0.14–0.66)	**0.010**	0.36	(0.17–0.78)
Frailty^2^	0.737	0.93	(0.59–1.45)			
Colostomy v ileostomy	0.355	0.85	(0.61–1.20)			
Length of stay (index procedure)	0.670	1.00	(0.99–1.01)			
30-day morbidity (index procedure)	0.415	1.15	(0.82–1.61)			
CD ≥ 3 (index procedure)	0.839	1.02	(0.84–1.24)			

Bold indicates p-value < 0.05; CI = Confidence Interval; BMI = Body Mass Index; SIMD = Scottish Index of Multiple Deprivation

1–Most deprived SIMD quintile (SIMD 1) compared to least deprived SIMD quintile (SIMD 5);

2–Frailty as indicated by a score of > 4 of the Rockwood Clinical Frailty Scale

Two significantly associated variables were identified in the final model. Male sex was associated with an increased time to stoma reversal (adjusted hazard ratio (HR) 0.69, 0.49–0.97, p = 0.032), with a median time to stoma reversal for males of 18.2 months compared to 14.5 months for females. Receiving adjuvant therapy for malignancy post-stoma formation was also associated with a delay to stoma reversal (adjusted HR 0.36, 0.17–0.78, p = 0.010). The presence of malignancy alone was not associated with delayed time to reversal (HR 1.11, 0.90–1.36, p = 0.330). Increasing BMI had a statistically significant relationship initially but not when taken forward to the final adjusted multi-variate Cox proportional-hazards model (adjusted HR 1.03 (0.99–1.06), p = 0.076).

### Reasons for non-reversal

**Fig. 4:**
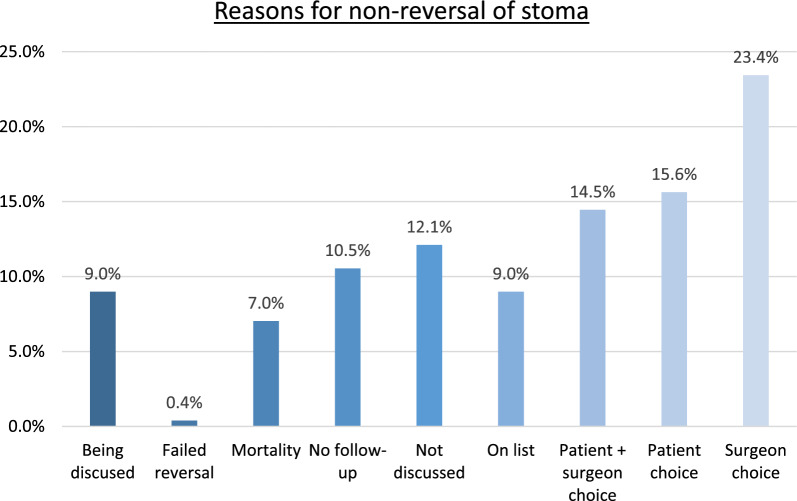
Reasons for non-reversal of a stoma

The reasons given for non-reversal of an emergency stoma can be seen in Fig. 4. A total of 53.5% of the non-reversed cohort opted for this after a documented discussion in an out-patient setting with their surgeon. 22.6% of patients of the non-reversed cohort either had no post-operative out-patient follow-up, or reversal of their stoma was not discussed in clinic. 9% of patients are currently on an elective waiting list for stoma reversal, and the same percentage are currently considering their options with regards to reversal. 7% of the non-reversed cohort died during follow-up (i.e. more than 1 year post-operatively).

### Factors associated with non-reversal

The results of the multi-variate logistic regression model for factors associated with non-reversal of an emergency stoma can be seen in Table 5. All factors identified as statistically significant in the univariate model remained so in the multi-variate model, with the exception of malignancy (Adjusted odds ratio (OR) 1.67, 0.96—2.91; p = 0.07).

**Table 5:** Logistic regression analysis for factors associated with non-reversal of an emergency stoma

Univariate analysis				Multi-variate analysis		
Variable	p-value	Odds Ratio	95% CI	p-value	Adjusted Odds Ratio	95% CI
Age	**0.009**	1.024	(1.006–1.043)	** < 0.001**	1.032	(1.016–1.048)
Sex (Male v Female)	0.998	1.001	(0.627–1.596)			
Deprivation^1^	**0.049**	1.718	(1.001–3.150)	**0.020**	2.119	(1.127–3.985)
Immuno-suppressed	**0.005**	2.507	(1.314–4.784)	** < 0.001**	2.677	(1.494–4.795)
CVD	0.695	0.899	(0.527–1.532)			
Diabetes	0.807	1.089	(0.549–2.163)			
Respiratory disease	0.767	0.919	(0.526 -1.606)			
Smoker	0.439	1.142	(0.544–2.398)			
ASA ≥ 3	** < 0.001**	2.740	(1.503–4.994)	**0.006**	2.405	(1.285–4.500)
BMI	0.321	0.981	(0.945–1.019)			
Malignancy	**0.048**	1.645	(1.005–2.693)	0.070	1.671	(0.958–2.914)
Adjuvant treatment	0.353	1.570	(0.606–4.068)			
Frailty^2^	**0.002**	1.902	(1.214–2.981)	**0.003**	2.029	(1.275–3.227)
Colostomy v ileostomy	0.205	0.733	(0.454–1.185)			

Bold indicates p-value < 0.05; CI = Confidence Interval; BMI = Body Mass Index; SIMD = Scottish Index of Multiple Deprivation

1–Most deprived SIMD quintile (SIMD 1) compared to least deprived SIMD quintile (SIMD 5);

2–Frailty as indicated by a score of > 4 of the Rockwood Clinical Frailty Scale

Non-reversal was more common in those living with frailty (adjusted OR 2.03, 1.28–3.23; p = 0.003), deprivation (adjusted OR 2.12, 1.13–3.99; p = 0.020), immunosuppression (adjusted OR 2.67, 1.49–4.80; p < 0.001), or for those who had an of ASA ≥ 3 (adjusted OR 2.41, 1.29–4.50; p = 0.006). Increasing age was also a statistically significant variable related to non-reversal of an emergency stoma (adjusted OR 1.03, 1.02–1.05; p < 0.001).

## Discussion

This multi-centre retrospective cohort study provides a real-life representation of outcomes after reversal of an emergency stoma within the United Kingdom. We have demonstrated that intestinal stoma formation is commonly performed during emergency abdominal surgery, in 29% of cases within our cohort which is in keeping with the 37.5% previously reported by NELA [17]. It is likely that this study is an underestimate of all emergency stomas formed due to a small amount of patients being lost to follow-up, and patients having a laparoscopic defunctioning stoma as a standalone procedure not being included. Overall, having a clear understanding of outcomes associated with reversal of these stomas is vital for patients and clinicians.

In keeping with previous research, we have confirmed that a stoma formed under emergency conditions is unlikely to be reversed, with only 28.9% of these patients undergoing stoma reversal after long-term follow-up. It is possible that more stomas would be reversed if all patients had clinical follow up after their emergency surgery, as over 20% of non-reversed patients had no post-operative follow-up. The development of appropriate guidelines for emergency stoma patients may help to standardise this process and ensure equity of access for the emergency stoma cohort.

We have demonstrated that the complication profile for elective reversal of an emergency stoma is substantial, with over half of patients developing post-operative complications. We identified no significant differences in post-operative outcomes between stoma type for those undergoing reversal, contrasting with previous literature from the elective setting that has suggested better outcomes for ileostomy reversal patients [9, 10]. This difference may be explained by the fact that many patients undergoing ileostomy reversal after emergency formation still require a laparotomy in order to perform this. When possible during the index emergency stoma formation, it is generally recommended to bring both ends of bowel out through the trephine as a ‘double-barrelled stoma’ [28] as this may allow reversal of the stoma via the stoma aperture in the future potentially improving outcomes after this procedure [29]. However, this was performed for less than 20% of the patients within our cohort, and there are currently no standards of care recommending or auditing this.

We have demonstrated that the reversal of an intestinal stoma within the first 18 months after emergency formation is associated with better post-operative outcomes. These patients have shorter in-patient hospital admissions, likely accounted for by the quicker return of normal gut function, and are over 75% less likely to develop significant complications than those who have their stoma reversed after this time period. Similar results have been demonstrated in the elective setting, for example by the CLOSE-IT study [13], that identified that prompt reversal of a defunctioning ileostomy after elective rectal cancer surgery appeared to be safe and may reduce stoma-related complications.

From the small amount of work available from the emergency stoma cohort, a longer time-period to reversal does appear to be associated with poorer outcomes, particularly when considering reversal of an end colostomy. For example, Resio et al. [30] found an increased length of hospital stay and 90-day readmission rate when colostomy reversal was delayed by more than 6 months for their American cohort of patients with complicated diverticulitis. However, these patients were mostly an ASA of 1 or 2, with high levels of private medical insurance, and had a reported median time to reversal of 5 months, and therefore are unlikely to be representative of the current patient profile seen within the NHS in the United Kingdom.

The explanation underlying the superior outcomes seen with prompt stoma reversal is likely to be multi-factorial, but may be due to atrophy of the distal limb of bowel being reversed that develops over time secondary to disuse. It has previously been hypothesised that this can lead to a longer operative time for the reversal procedure, poorer anastomotic function and an increase in peri-operative complications [31, 32]. Given the improved outcomes demonstrated within our research, the authors recommend that an emergency stoma is reversed within 18 months of formation, whenever feasibly possible. However, future research could focus on the impact of stoma reversal even earlier than this for emergency patients, particularly focussing on the impact this may have on stoma-related complications and patient-reported outcome measures.

A further implication of this research is to provide support to the development of ‘hot’ (or acute) and ‘cold’ (or elective) operative sites within the NHS. This idea has previously been proposed as a strategy to combat the substantial elective surgical waiting lists within the NHS [33, 34]. The promotion of ‘cold’ operative sites within the UK could improve the median time to stoma reversal for emergency stoma patients, and subsequently may improve post-operative outcomes.

We have demonstrated that male patients and those receiving adjuvant treatment for malignancy are more likely to wait longer to have their stoma reversed, and that this is associated with poorer post-operative outcomes. Female sex has previously been shown to be a risk factor for poorer quality of life post-emergency stoma formation [35]. Therefore, this increased impact on quality of life may potentially mean that female patients are more determined to have their stoma reversed in a timely manner.

Several expected risk factors have been identified as being associated with non-reversal of an emergency stoma: increasing age; the presence of pre-operative frailty; and increasing ASA grade at index surgery [10, 25, 36]. However, the association of increasing socio-economic deprivation with non-reversal is a novel finding. The complex relationship underlying this may be partially explained by the reduced access to healthcare services seen for patients who are living with deprivation [37, 38], meaning that they are less likely to pro-actively seek or engage with surgical follow-up.

The findings of our study must be appraised considering some limitations. Firstly, no distinct protocol was available to direct surgeons or patients in the decision-making process underlying emergency stoma reversal. Thus, the decision was dependent on the individuals in each case, and this may have introduced some selection bias. However, given the current lack of guidance available for these discussions, this was felt to be a true to life representation for clinicians working within the NHS. Further exploration of the decision-making process underlying reversal for these patients and development of guidelines for this would be beneficial.

Secondly, the patients in our cohort were identified between 2018 and 2021, with patients being followed up until 2024. Clearly the impact of the COVID-19 pandemic on elective surgical services during this period was substantial, and may have resulted in patients waiting longer for elective procedures such as stoma reversal. However, the lack of elective operating capacity associated with the COVID-19 pandemic does not entirely explain the relatively low rates of stoma reversal within this cohort. This is demonstrated by the subjective reasoning for non-reversal shown in Fig. 4, showing that only 9% of the non-reversed patients in our cohort are currently on a waiting list to have their stoma reversed. If lack of elective capacity was the sole explanation for this, then this figure would have been presumed to have been much higher. This indicates that there may be more diverse reasons underlying the relatively low reversal rate within our cohort, the impact of which may be a focus for future longitudinal research.

And finally, this study involved retrospective analysis of prospectively maintained data. Therefore, data analysis is reliant on the quality and accuracy of real time data collection by various individuals at the four included sites in the first instance.

## Conclusion

An intestinal stoma is commonly created during emergency abdominal surgery and is associated with a high mortality rate. Less than a third of emergency stomas are reversed, and those patients that undergo reversal are subject to a substantial complication profile, that is reduced if reversed early. There is a clear clinical need to optimise and standardise the pathway for elective stoma reversal across the UK.

## Data Availability

The datasets generated during and/or analysed during the current study are available from the corresponding author on reasonable request.

## Abbreviations

ASA: American Society of Anaesthesiologists
BMI: Body Mass Index
EmLap: Emergency laparotomy
ELLSA: Emergency Laparotomy and Laparoscopic Scottish Audit
HR: Hazard ratio
IQR: Inter-quartile range
NBOCA: National Bowel Cancer Audit
NELA: National Emergency Laparotomy Audit
NHS: National Health Service
OR: Odds ratio
SIMD: Scottish Index of Multiple Deprivation
SD: Standard deviation
SRCs: Stoma-related complications
UK: United Kingdom
